# Extensive Cholesteatoma Compromising the Entire Ipsilateral Skull Base: Excision Through a Multi-Corridor Surgical Technique

**DOI:** 10.3390/reports8030148

**Published:** 2025-08-18

**Authors:** Lyubomir Rangachev, Julian Rangachev, Tzvetomir Marinov, Sylvia Skelina, Todor M. Popov

**Affiliations:** 1 Department of ENT, Medical University of Sofia, 1431 Sofia, Bulgaria; lyubomir.rangachev@gmail.com (L.R.);; 2 Department of Anesthesiology, Medical University of Sofia, 1431 Sofia, Bulgaria; 3 Department of Neurology, Medical University of Sofia, 1431 Sofia, Bulgaria

**Keywords:** transsphenoid and transclival approach, retrolabyrinthine approach, massive cholesteatoma, skull base

## Abstract

**Background and Clinical Significance**: Petrous bone cholesteatoma is a rare and complex condition that poses significant challenges in terms of its diagnosis and treatment. This benign yet locally aggressive lesion can cause extensive destruction of the surrounding structures, potentially leading to serious complications. **Case Presentation**: We present a case of extensive petrous bone cholesteatoma involving nearly the entire skull base. High-resolution CT and MRI were used to assess the extent of the lesion and its relationship with critical neurovascular structures. The cholesteatoma extended from the petrous apex to the clivus, involving the internal auditory canal and Meckel’s cave, encasing the internal carotid artery, and compressing the brainstem. The surgical strategy included combined endoscopic transsphenoidal and transclival techniques with a retrolabyrinthine approach. The endoscopic component provided access to the anterior and central skull base regions, whereas the retrolabyrinthine approach allowed us to gain access to the posterior petrous area. Careful dissection was performed to separate the cholesteatoma from the internal carotid artery and the brainstem. Neuromonitoring was performed throughout the procedure to ensure cranial nerve integrity. This combined approach enabled gross total resection, and postoperative imaging confirmed successful tumor removal. The patient’s recovery was uneventful, and no new neurological deficits were observed. **Conclusions**: The successful management of this complex case demonstrates the efficacy and safety of combining endoscopic surgical approaches for extensive skull base cholesteatomas. This multi-corridor approach allows for maximal tumor resection while also minimizing the risks to critical neurovascular structures.

## 1. Introduction and Clinical Significance

Cholesteatoma, a well-recognized pathological condition among otolaryngologists, is characterized by the accumulation of keratinizing squamous epithelium, which induces a tissue reaction. Non-neoplastic cholesteatomas are proliferative and destructive, leading to symptoms associated with the disruption of the function of adjacent structures. They predominantly affect the middle ear and mastoid cavity, with only approximately 3% occurring primarily in the petrous bone, accounting for 3–9% of all temporal bone lesions [1]. The most frequently employed classification system, proposed by Sanna et al., categorizes petrous bone cholesteatoma (PBC) into five classes, supralabyrinthine (49–70%), infralabyrinthine (7–12%), massive (5–38%), infralabyrinthine-apical (5–7%), and apical (1–5%), with three subclasses: clival, sphenoid, and rhinopharyngeal [1,2]. Common surgical approaches for the treatment of PBC include the translabyrinthine, subtotal petrosectomy, middle fossa-transmastoid, infratemporal type B, and transcochlear approaches [1,2] (Table 1). In this report, we present an extremely rare case of a 72-year-old patient with recurrent massive PBC, which practically destroyed the entire ipsilateral skull base, treated using a combined transnasal transclival endoscopic approach and a retrolabyrinthine approach in two stages.

## 2. Case Presentation

### 2.1. History

A 72-year-old man presented with fever (39.9 °C), grade 6 facial palsy on the House–Brackmann scale, nausea, an unstable gait, left-sided paresthesia, transient diplopia, and a headache localized to the temporo-auricular–parieto-occipital region, all of which were managed solely with antipyretics. The patient reported experiencing progressive facial asymmetry and reduced hearing 17 years prior. Two years later, he underwent two successive surgeries for cholesteatoma, the first of which was conducted by an ENT specialist, while the second took place two months later in the neurosurgical department. An otologic examination revealed no signs of otorrhea or local recurrence in either patient. A review of his medical records indicated a diagnosis of PBC, initially treated surgically with wall-up mastoidectomy, facial nerve decompression, and tympanoplasty, followed by a temporal craniotomy with the visually radical extirpation of a subtemporal, extradural cholesteatoma, which had caused significant destruction of the pyramid. Upon admission, antibiotic treatment was initiated, and a head CT revealed a petrous bone lesion involving the clivus and sphenoid sinus, along with extradural pneumocranium. Pure-tone bone conduction thresholds revealed unilateral hearing loss equal to 30–50 dB HL in the right ear, indicative of mild to moderate sensorineural hearing loss, while the left ear exhibited anacusis. The presence of a dead labyrinth was confirmed through audiometric evaluation and imaging, attributed to the effects of cholesteatoma and prior surgical intervention. Based on presurgical MRI data, the lesion was classified as apical PBC, extending to Meckel’s cave area and the horizontal ICA anteriorly, the IAC and posterior cranial fossa posteriorly, and the clivus and cavernous segment of the ICA medially. As a result of complete extirpation with the proposed fibrin glue repair of the petrous defect, extensive scarring and adhesions to the ICA were anticipated.

### 2.2. CT Imaging and Surgical Planning

**Figure 1:**
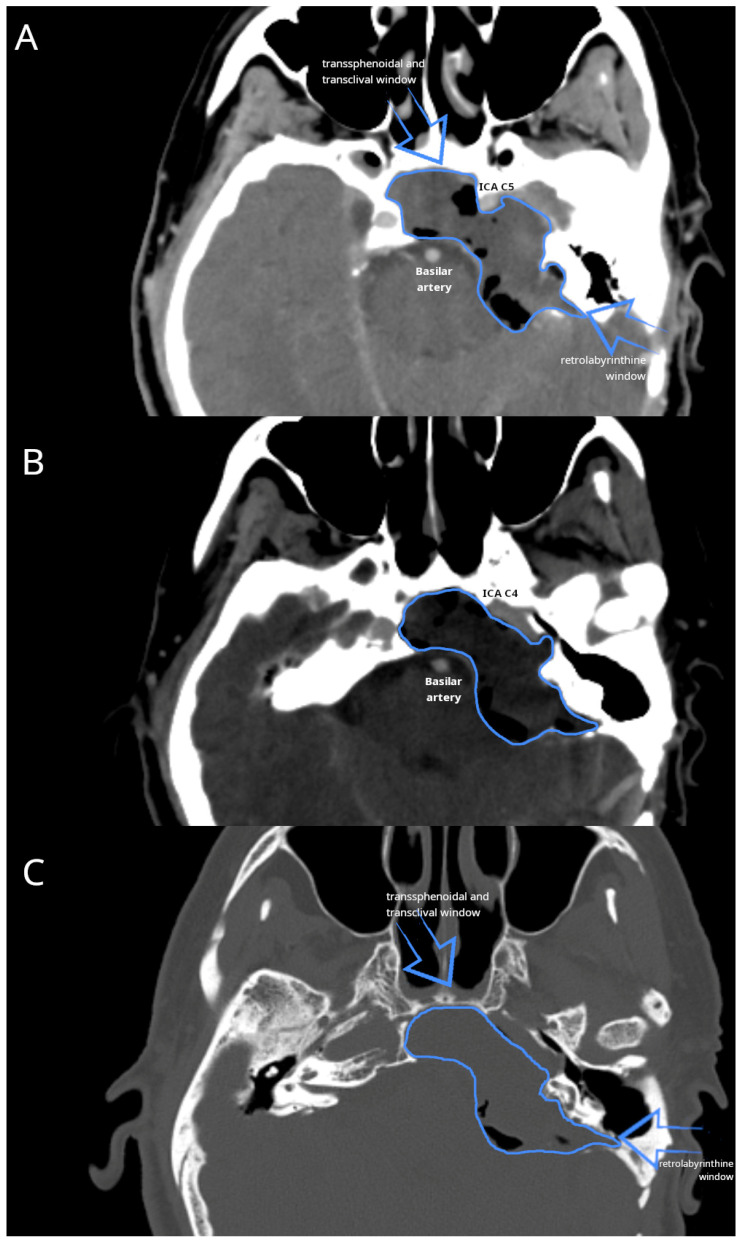
Preoperative CT imaging of the lesion. ICA (internal carotid artery), basilar artery, and cholesteatoma (encircled in blue) are indicated. (**A**,**B**) CT angiography; (**C**) high-resolution CT image.

High-resolution CT imaging of the temporal bone, in conjunction with CT angiography, was used to delineate the anatomical relationships of the structures. The cholesteatoma extended from the petrous apex to the clivus, involving the internal auditory canal and Meckel’s cave, encasing the internal carotid artery, and compressing the brainstem. The formation closely adhered to the ICA along its C2-C4 segments anteriorly and to the sigmoid sinus and cerebellopontine dura posteriorly, without involving the jugular bulb (Figure 1).

Based on these findings and the patient’s previous surgical history, the lesion was suspected to be a recurrence of the cholesteatoma. We decided to employ a transnasal endoscopic approach to access the paraclival area. This approach has been demonstrated to be both effective and safe for the treatment of neoplastic diseases in this region [3]. There are documented cases of cholesterol granulomas of the petrous apex being drained [4] and PBC being managed through resection [5,6] or drainage [7] with a long-term follow-up. Utilizing a medial approach to the ICA and paraclival space reduces the risk of arterial tearing associated with mobilization during the lateral approach. To address the posterolateral extension of the PBC, a second-stage transmastoid surgery was planned [8].

### 2.3. Two-Stage Surgical Procedure

#### 2.3.1. Transnasal Transclival Endoscopic Approach

**Figure 2:**
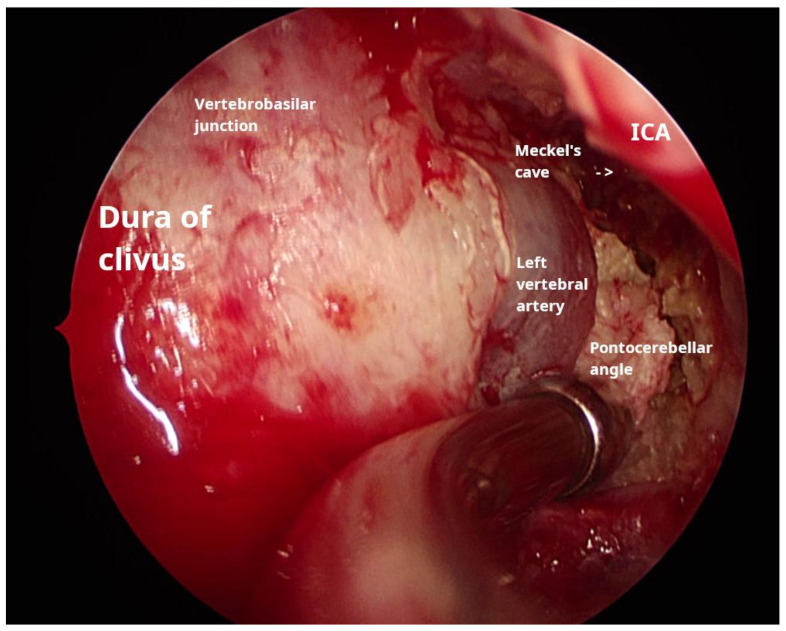
Intraoperative endoscopic view of the trans-sphenoid and transclival approach along with annotated structures.

During the procedure, the anterior wall of the right ethmoid bulla was excised; then, an antrostomy was carried out. In the absence of a retrobullar recess, access to the posterior ethmoid was achieved through the posterior wall of the bulla, specifically the basal lamella of the middle turbinate. Utilizing the inferomedial triangle approach, as delineated by Bolger et al. [9], entry into the sphenoid sinus was accomplished, where a yellowish, greasy, layered matrix was encountered. The matrix was removed using suction and irrigation, allowing for visualization of the sellar prominence, followed by the carotid prominence on the right side. Further excision of the cholesteatoma towards the clival recess revealed a bone defect through which the dura was visible. To increase visibility, the floor of the sinus wall was removed, and a posterior septectomy was conducted. Adjacent to the clival defect, the intersinus septa was posteriorly connected to the remnants of the left-side carotid prominence; careful bone removal facilitated visualization of the left carotid artery. Further lateral excision of the cholesteatoma matrix exposed pus-filled cavities and revealed the dural sleeve of the abducens nerve. Debulking continued until Meckel’s cave was visualized. Matrix removal proceeded in a posterolateral direction until the suction tip could no longer access additional material, and the medial aspect of the internal auditory canal (IAC) was palpated. Careful dissection was performed to separate the cholesteatoma from the internal carotid artery and the brainstem (Figure 2, Video S1). Neuromonitoring was conducted throughout the procedure to ensure the integrity of the cranial nerves.

#### 2.3.2. Retrolabyrinthine Approach

Two months after the initial procedure, a transmastoid canal-wall-down operation was performed on the left side. This involved the excision of the cholesteatoma matrix extending from the sigmoid sinus posterolaterally to the internal auditory canal medially utilizing a retrolabyrinthine approach. Additionally, transtegmental access to the middle cranial fossa was achieved, facilitating the removal of the material inferior to the dura of Meckel’s cave. By keeping the cavity open through the external auditory canal, we ensured potential drainage, as the extradural space was exteriorized via the nasal approach. This combined approach enabled gross total resection, and postoperative imaging confirmed successful tumor removal.

### 2.4. Outcome and Follow-Up

In the initial period following transnasal surgery, the patient experienced an improvement in their headache and nausea symptoms. Upon the discontinuation of antibiotic treatment, no fever or pain was observed, and all signs of acute inflammation had resolved. After the second intervention, only minor wound secretions were noted, which diminished during the postoperative period following the administration of levofloxacin ear drops, despite the absence of strong evidence supporting its efficacy [10].

**Figure 3:**
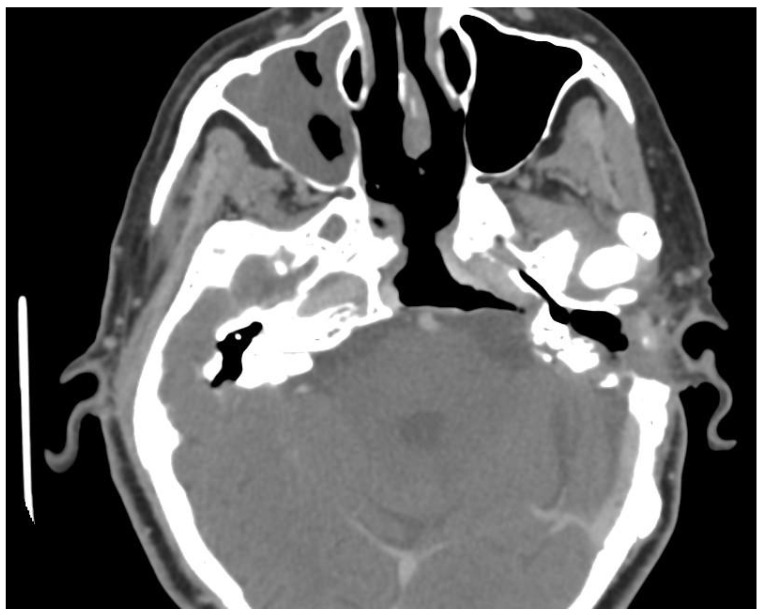
Postoperative CT imaging of the skull base, with no detectable recurrence.

During the brief follow-up period (lasting two months), the ear cavity underwent epithelialization without any indications of inflammation or exudate retention. However, there was noticeable crusting within the sphenoid sinus cavity, accompanied by epidural exudate retention. The patient experienced a transient period of nasal obstruction and mucus secretion that resolved spontaneously. No recurrence of presurgical symptoms has been reported since. A high-resolution CT scan with contrast enhancement, conducted four months post-transnasal surgery to facilitate improved visualization of the vascular structures, revealed a wide resection of the cholesteatoma and no signs of recurrence or infection in the surgical cavity (Figure 3).

### 2.5. Complications

On the second postoperative day following transsphenoidal surgery, the patient experienced dyspnea, leading to suspicion of acute pulmonary embolism, which was subsequently confirmed via pulmonary angiography. The patient was then transferred to the cardiology department within the same hospital, where he received treatment and was discharged with improvement noted after five days. In the fourth postoperative month, the patient reported the occurrence of clear fluid dripping from the nose during exertion, but without the typical postural headache. Flexible endoscopic examination of the postsurgical cranial base defect revealed no signs of cerebrospinal fluid (CSF) leakage. As this symptom was recently reported, we have not yet been able to collect samples for beta-2 transferrin testing [11].

## 3. Discussion

Conducting regular follow-ups is essential for patients treated for cholesteatomas because of the relatively high recurrence rates associated with this non-neoplastic condition. Monitoring after treatment of PBC presents an even greater challenge [12]. Our decision to employ staged surgical approaches was based on the necessity of radical intervention in an area where comprehensive visualization of the formation was unattainable. This strategy proved advantageous in resecting the formation from structures such as the ICA and sigmoid sinus, providing a safer and more convenient point of access, thereby significantly enhancing our control over the procedure. Given the tumor’s substantial size, dissemination, and proximity to numerous critical structures, these surgical corridors afforded us an optimal opportunity to visualize and excise the pathology comprehensively without compromising these structures. Faced with systemic complications arising from this advanced local disease, our primary objective was to address these issues and prevent further progression. Our aims included decompressing the cranial cavity and resolving the inflammatory process. The combination of approaches employed facilitated GRT, with subtotal resection being considered a favorable outcome if symptoms were alleviated. We acknowledge the limitations posed by the short follow-up period and the yet undefined, but possible, occurrence of nasal liquorrhea. Nevertheless, based on its demonstrated efficacy in addressing skull base neoplastic disease, the multimodal endoscopic approach is a valuable addition to the repertoire of surgical techniques for the management of PBC. Regarding the reported complications, timely interdisciplinary collaboration was crucial for the successful resolution of acute issues.

## 4. Conclusions

Involvement of the petrous bone is a rare and challenging manifestation of cholesteatoma. Imaging studies facilitate the planning of the most appropriate surgical approach to treating this condition. The combination of endoscopic trans-sphenoidal and transclival approaches, along with the retrolabyrinthine approach, has been demonstrated to be an effective and safe method for resecting tumors that invade nearly the entire skull base.

## Figures and Tables

**Table 1:** Summary of the most commonly used approaches to treating PBC.

Approach	Lesion Location	Procedure Includes	Advantages	Disadvantages
Subtotal petrosectomy	Petrous bone	Canal-wall-down tympanomastoidectomy, fallopian canal skeletonization, and surgical cavity obliteration	It enables wide access to the petrous apex and poses a lower risk of a cerebrospinal fluid (CSF) leak compared to other, similar approaches	It entails hearing loss and a risk of damage to the facial nerve, internal carotid artery (ICA), or jugular bulb
Middle fossa approach	Internal auditory canal (IAC), petroclival region, prepontine cisterns, and upper to middle clivus in extended approach	Craniotomy, elevation of dura and localization of landmarks (arcuate eminence and greater superficial petrosal nerve), IAC skeletonization, and nerve identification	It preserves hearing and is ideal for smaller-tumor resection	It facilitates limited exposure, entails temporal lobe retraction, and poses a risk of damage to the facial nerve
Translabyrinthine approach	IAC and cerebellopontine angle (CPA)	Mastoidectomy, posterior cranial fossa bone removal, labyrinthectomy, and skeletonization of the IAC	It offers a direct path to the IAC and allows access to the facial nerve along its segments	It entails hearing loss, CSF leakage and is hard to perform in the case of some anatomical variations (low tegmen, anterior sigmoid sinus, and high jugular bulb)
Transotic approach	CPA anterior to the IAC and petrous apex	Canal-wall-down tympanomastoidectomy, labyrinthectomy, fallopian canal, ICA and jugular bulb skeletonization, and surgical cavity obliteration	It allows near circumferential access to the IAC and porus and has a better chance of preserving the facial nerve	It facilitates reduced exposure compared to the transcochlear approach and poses a risk of ICA damage and possible CSF leakage
Transcochlear approach	Petrous apex, CPA, and clivus	Canal-wall-down tympanomastoidectomy, labyrinthectomy, drilling out of the cochlea and fallopian canal with rerouting of the facial nerve, ICA and jugular bulb skeletonization, and surgical cavity obliteration	It allows wide CPA exposure without brain retraction and grants access to CN V-XI and the vertebral and basilar arteries	It sacrifices residual hearing, poses a high risk of facial nerve damage, poses a risk of ICA damage, and potentially leads to CSF leakage
Infratemporal fossa approach type B	Petrous apex and clivus, petrous ICA, inferior temporal surface, and CN V-XII	Transection and closure of the EAC, division of the zygomatic arch, removal of the floor of the skull base, skeletonization of the petrous ICA, detachment of the eustachian tube, and obliteration of the surgical cavity	Enables wide exposure of lateral skull base	It entails conductive hearing loss, facial nerve damage, and possible mandible dislocation
Endoscopic transsphenoidal transclival approach	Sphenoid sinus, sellar and parasellar region, clivus, ventral brainstem, and craniovertebral junction	Turbinatectomy, antrostomy, posterior ethmoidectomy, removal of the inferior sphenoid sinus wall, posterior septectomy, and drilling of the clivus	It is less invasive, potentially enables reconstruction with pedicled flaps, allows multilevel exposure (sellar, clival, and craniovertebral), and involves less manipulation of neurovascular structures	It poses a risk of CSF leakage, allows only limited lateral exposure, and requires training in skull base surgery

## Data Availability

The original contributions presented in this study are included in the article/Supplementary Materials. Further inquiries can be directed to the corresponding author.

## Supplementary Materials

The following supporting information can be downloaded at https://drive.google.com/file/d/1gYtDP1PCHCkyhHb-h9mD8zhZoBNReKCc/view?usp=drive_link. Video S1: Extensive cholesteatoma compromising the entire ipsilateral skull base: excision through a multi-corridor surgical technique.

## Abbreviations

The following abbreviations are used in this manuscript:

PBC	Petrous bone cholesteatoma
IAC	Internal auditory canal
CSF	Cerebrospinal fluid
ICA	Internal carotid artery
CPA	Cerebellopontine angle
CT	Computer tomography
MRI	Magnetic resonance imaging

## References

[1] Sanna M., Pandya Y., Mancini F., Sequino G., Piccirillo E. (2011). Petrous Bone Cholesteatoma: Classification, Management and Review of the Literature. Audiol. Neurotol..

[2] Danesi G., Cooper T., Panciera D.T., Manni V., Côté D.W.J. (2016). Sanna Classification and Prognosis of Cholesteatoma of the Petrous Part of the Temporal Bone. Otol. Neurotol..

[3] Frank G., Sciarretta V., Calbucci F., Farneti G., Mazzatenta D., Pasquini E. (2006). The Endoscopic Transnasal Transsphenoidal Approach for the Treatment of Cranial Base Chordomas and Chondrosarcomas. Oper. Neurosurg..

[4] Georgalas C., Kania R., Guichard J.-P., Sauvaget E., Tran Ba Huy P., Herman P. (2008). Endoscopic transsphenoidal surgery for cholesterol granulomas involving the petrous apex. Clin. Otolaryngol..

[5] Aubry K., Kovac L., Sauvaget E., Huy P.T.B., Herman P. (2010). Our experience in the management of petrous bone cholesteatoma. Skull Base.

[6] Xu R., Zhang Q.H., Zuo K.J., Xu G. (2012). Resection of petrous apex cholesteatoma via endoscopic trans-sphenoidal approach. Zhonghua Er Bi Yan Hou Tou Jing Wai Ke Za Zhi Chin. J. Otorhinolaryngol. Head Neck Surg..

[7] Nishida N., Fujiwara T., Satoshi S., Inoue A., Takagi D., Takagi T., Hato N. (2021). Exteriorization of Petrous Bone Cholesteatoma by Endonasal Endoscopic Approach: A Case Report. J. Int. Adv. Otol..

[8] Morino T., Yamamoto Y., Yamamoto K., Komori M., Asaka D., Kojima H. (2021). Management of Intractable Petrous Bone Cholesteatoma with a Combined Translabyrinthine-Transsphenoidal Approach. Otol. Neurotol..

[9] Bolger W.E., Keyes A.S., Lanza D.C. (1999). Use of the Superior Meatus and Superior Turbinate in the Endoscopic Approach to the Sphenoid Sinus. Otolaryngol. Head Neck Surg..

[10] Verschuur H.P., de Wever W.W., van Benthem P.P. (2004). Antibiotic prophylaxis in clean and clean-contaminated ear surgery. Cochrane Database Syst. Rev..

[11] Eide J.G., Mason W., Mackie H., Cook B., Ray A., Asmaro K., Robin A., Rock J., Craig J.R. (2025). Diagnostic Accuracy of Beta-2 Transferrin Gel Electrophoresis for Detecting Cerebrospinal Fluid Rhinorrhea. Laryngoscope.

[12] Touska P., Connor S.E.J. (2025). ESR Essentials: Imaging of middle ear cholesteatoma-practice recommendations by the European Society of Head and Neck Radiology. Eur. Radiol..

